# The triglyceride–glucose index is a promising predictor for the risk of cardiovascular disease in the diabetic population aged ≥60 years in the United States: a retrospective cohort study from NHANES (2007-2016)

**DOI:** 10.3389/fendo.2025.1475590

**Published:** 2025-02-21

**Authors:** Shu Yang, Zhenwei Wang

**Affiliations:** ^1^ School of Medicine, Jiangsu University, Zhenjiang, China; ^2^ Department of Cardiology, The First Affiliated Hospital of Zhengzhou University, Zhengzhou, China

**Keywords:** triglyceride-glucose index, cardiovascular disease, diabetes mellitus, elderly people, NHANES

## Abstract

**Background:**

The predictive value of triglyceride-glucose index (TyG) for cardiovascular disease (CVD) in the US elderly diabetic patients is ambiguous. This study aimed to investigate the association between TyG index and the risk of CVD in an older US population with diabetes.

**Methods:**

The study examined data from the 2007-2016 National Health and Nutrition Examination Survey (NHANES). Univariate and multivariate regression analysis models were obtained to explore the association between baseline TyG index and the risk of CVD. Non-linear association were investigated using restricted cubic spline (RCS) regression. Subgroup analyses and interaction tests were constructed and a sensitivity analyses was carried out. The 10 - year CVD risk were evaluated via the Framingham Risk Score (FRS). Mediation analysis explored the mediating role of glycated hemoglobin in the above relationships.

**Results:**

A total of 2987 subjects were included (977 CVD patients and 2010 non-CVD persons). CVD patients had higher TyG values (9.01 ± 0.58 vs. 8.94 ± 0.56, P=0.003), and the prevalence of CVD increased with TyG index (P=0.015). In a multifactorial regression model with gradual adjustment for all covariates, the risk of CVD associated with TyG increased by 48.0% in the highest quartile group (OR 1.480, 95% Cl 1.171-1.871, P=0.001). The RCS curves showed a U-shaped association between TyG index and CVD risk (P for overall=0.013, P for nonlinear=0.043). Subgroup analyses showed that in the highest quartile group, individuals with body mass index (BMI) ≥24 kg/m^2^, an estimated glomerular filtration rate (eGFR) <90 mL/1.73m^2^/min, individuals without chronic kidney disease, and those with hypertension had significantly higher risks of CVD. Sensitivity analyses indicated that these associations were not associated with other significant confounders. Under different adjustment models, the TyG index exhibited significant correlations with the 10 - year risk of CVD (all P values < 0.05). Glycated hemoglobin mediated in the above relationships.

**Conclusion:**

In a sample of US elderly diabetic patients, there is the U-shaped association of TyG index with CVD risk. This implies that TyG index can be regarded as an extremely important predictor for screening people at high risk of cardiovascular disease among elderly diabetic patients.

## Background

Cardiovascular disease (CVD) is regarded as one of the most seriously burdened health problems globally, and the latest epidemiologic reports of its prevalence are increasing every year (1, 2). It is worth noting that type 2 diabetes is prevalent among patients diagnosed with CVD and is associated with adverse outcomes (3). Therefore, early screening is of vital importance for the risk of cardiovascular death in diabetic patients to be reduced.

As a surrogate marker of insulin resistance derived from routine indicators, the triglyceride-glucose index (TyG) is more cost - effective, convenient and sensitive. It has been developed as a biochemical substitute for identifying insulin resistance in both diabetic and non - diabetic patients (4, 5). Numerous studies indicate that the TyG index serves as an independent predictor of future cardiovascular mortality and other subclinical cardiovascular diseases in the general population (6–8). Meanwhile, related research has confirmed the certain clinical value of the TyG index in relation to adverse cardiovascular events among both non - diabetic and diabetic patients (9). However, among diabetic patients of different age groups, the conclusion about using the TyG index to predict CVD risk remains controversial. Particularly in elderly diabetic patients, physical decline, co - morbidity of multiple chronic diseases, and intricate drug interactions alter their insulin - resistance patterns, blood - glucose fluctuation characteristics, as well as the structure and function of the cardiovascular system (10, 11). These result in differences in CVD pathogenesis and risk levels compared to other populations. Currently, targeted research in this regard is scarce.

Therefore, our study aimed to explore the association between the TyG index and the risk of CVD in elderly patients with diabetes, evaluate whether the TyG index has prognostic value for CVD risk in this population, and uncover the non - linear relationship between them.

## Methods

### Study population

**Figure 1:**
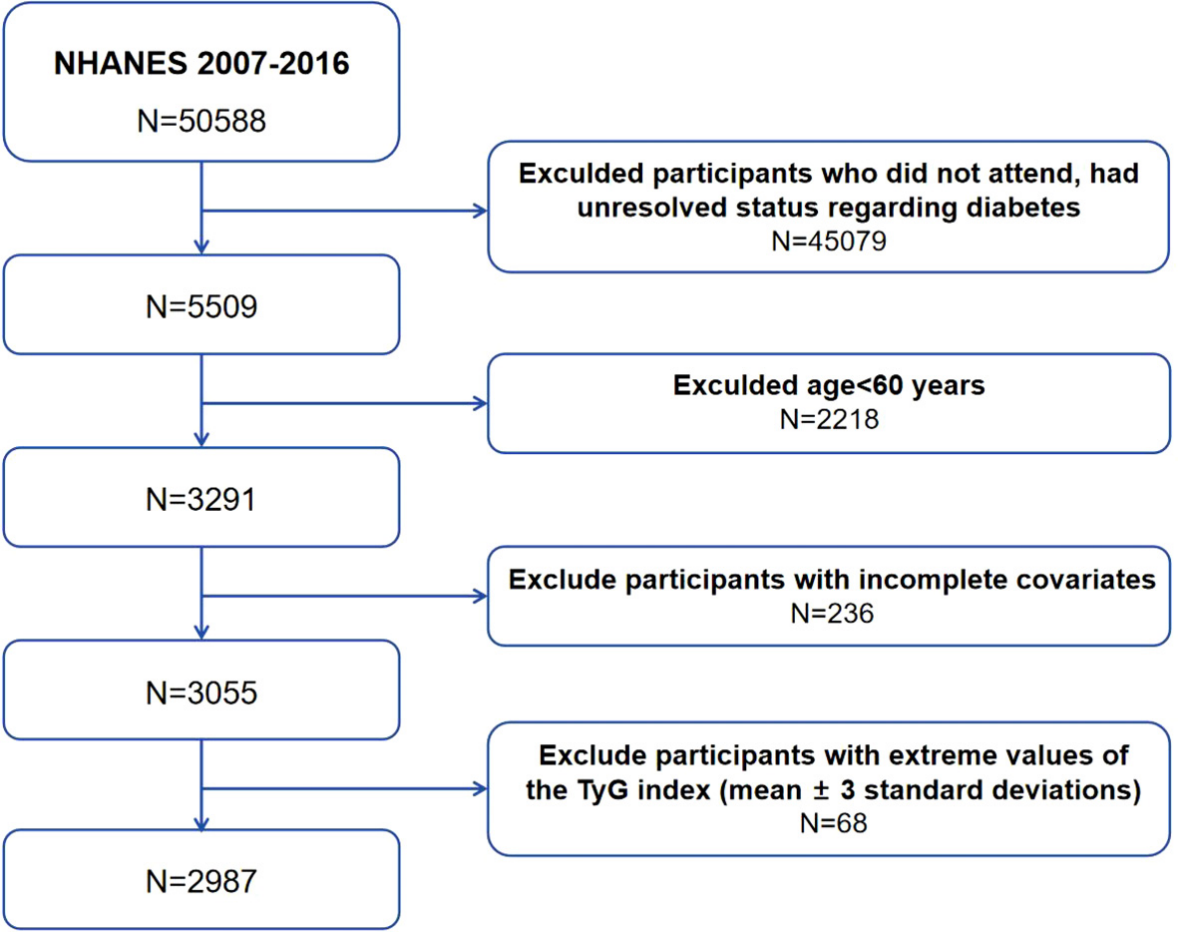
Flowchart of participants’ selection. National Health and Nutrition Examination Survey (NHANES), The triglyceride-glucose index (TyG).

National Health and Nutrition Examination Survey (NHANES) is a research program led by the National Center for Health Statistics (NCHS) of the CDC dedicated to measuring the health and nutritional status of Americans, adults, and children. Through integrated interviews and physical exams, it yields valuable insights on prevalent diseases, dietary and nutritional trends, and health-related behaviors, serving as a premier database for probing disease epidemiology and related risk factors. All participants provided written informed consent before participation in the survey. The data in this study were anonymized and de-identified before the analysis to ensure the privacy and confidentiality of the participants. In this retrospective observational study, a total of 50,588 participants were initially enrolled. Participants who did not attend, had unresolved status regarding diabetes (n=45,079), were under the age of 60 (n=2,218), or for whom the covariates were incomplete (n=236) were excluded. Participants with extreme values of the TyG index (mean ± 3 standard deviations) were also excluded (n=68). Ultimately, a total of 2987 participants with complete data were incorporated into this analysis (**Figure 1**).

### Assessment of covariates

Demographic data included age, gender, race, education level, and marital status. Race was categorized as Mexican American, non-Hispanic black, non-Hispanic white, other Hispanic, and other races; education level was categorized as less than high school, high school or equivalent, and college or above; and marital status was categorized as married, widowed, divorced, and never married. Questionnaire data included smoking status, alcohol consumption, and medication use (insulin, hypoglycemics, antihypertensive drugs), etc. The smoking and alcohol consumption statuses were recorded as “yes” if the subjects agreed to smoke at least 100 cigarettes in a lifetime and drink at least 12 alcoholic per year, respectively. Blood pressure and body measurements were obtained from the examination data. Blood pressure were recorded as the maximum measurement, or the final value if there was only one measurement; body mass index (BMI) was calculated as weight (kg)/height (m) squared. In laboratory data, data on fasting blood glucose (FBG), glycated hemoglobin (HbA1c%), total cholesterol (TC), triglyceride (TG), high-density lipoprotein cholesterol (HDL-C), low-density lipoprotein cholesterol (LDL-C), and albumin and uric acid were collected. For more detailed measurement data, please visit the NHANES website.

### Data definitions

Diabetes mellitus was defined as satisfying any one of the following conditions: (1) FBG ≥ 7 mmol/L; (2) Random blood glucose or 2-hour oral glucose tolerance test (OGTT) ≥ 11.1 mmol/L; (3) HbA1c ≥ 6.5%; and (4) Answering “yes” to one of the following questions: “Your doctor has ever told you that you have diabetes” “You are now taking insulin” “You are now taking hypoglycemic drugs”. Subjects were considered to have CVD if they answered “yes” to the question “Have you ever suffered from congestive heart failure (CHF)/coronary heart disease (CHD)/angina/myocardial infarction/stroke” by a doctor or other health specialist. Hypertension was defined as being informed of hypertension or use of antihypertensive drugs or SBP ≥ 140 mmHg or DBP ≥ 90 mmHg. Chronic kidney disease (CKD) was determined by answering “yes” to the question, “Have you ever been told you have kidney failure?” or by calculating an estimated glomerular filtration rate (eGFR) of < 60 mL/1.73m^2^/min, which was proposed based on the 2021 Chronic Kidney Disease Epidemiology Collaboration (CKD-EPI) (12).

### Definitions of triglyceride-glucose index

TyG index as an exposure variable was calculated by the following formula: TyG = Ln[TG (mg/dL)*FBG (mg/dL)/2]. TyG index was divided into four groups, i.e., Q1 (≤ 8.67) 752 cases, Q2 (8.67-8.96) 729 cases, Q3 (8.96-9.20) 767 cases, and Q4 (≥ 9.20) 739 cases. Both triglyceride and fasting glucose concentrations were measured through an enzymatic assay using an automatic bio-chemistry analyzer. Serum triglyceride concentration was measured using the Roche Modular P and Roche Cobas 6000 chemistry analyzers while fasting plasma glucose was assessed through the hexokinase-mediated reaction using the Roche/Hitachi Cobas C 501 chemistry analyzer.

### Cardiovascular disease risk

We employed the Framingham Risk Score (FRS) to evaluate participants’ 10 - year CVD risk. This score gauges the 10 - year composite risk of coronary heart disease, stroke, peripheral arterial disease, and heart failure. The algorithm, based on age (years), sex (male/female), total and HDL cholesterol (mg/dL), SBP (mmHg), treated and untreated hypertension, smoking status (yes/no), and diabetes status (yes/no), calculates each participant’s CVD risk score per sex - specific criteria. This score is then translated into a 10 - year CVD risk, expressed as FRS%. Specific scoring details are in Tables 5 - 8 of the reference (13).

### Statistical analysis

Data in NHANES were sourced from the items in multiple sections, and inevitably some values are missing. In this study, the missing data issue was tackled via multiple imputation based on random forests. Through multiple samplings and predictions, we got imputed complete datasets, better preserving info, correcting biases, and boosting statistical power. Continuous variables were reported as mean ± standard deviation (SD) using the independent samples T test when they conformed to a normal distribution, and as median (25th-75th percentile) using the Mann-Whitney U test for non-normal distribution. Categorical variables were reported as frequencies and percentages using chi-square and Fisher’s exact tests. Besides, TyG index was divided the into quartile groups to compare their baseline characteristics. One-way analysis of variance (ANOVA), Kruskal-Wallis H test, and chi-square and Fisher’s exact tests were performed.

Univariate and multivariate logistic regression models were developed to investigate the relationship between TyG index and the risk of CVD at continuous and categorical variables. Model 1 was not adjusted for covariates. Model 2 was adjusted for age and sex. Model 3 was adjusted for age, sex, race, education level, marital status, smoking status, and DBP. Model 4 was adjusted for all covariates, including age, sex, race, education level, marital status, smoking status, DBP, TC, HDL-C, LDL-C, albumin, eGFR, uric acid, Hypertension, CKD, antihypertensive drugs. For subgroup analyses, the groups were separated by sex (male/female), BMI (< 24/≥ 24 kg/m^2^), eGFR (< 90/≥ 90 mL/1.73m^2^/min), CKD (Yes/No), and Hypertension (Yes/No). Ratio ratios (ORs) of the different strata were compared by interaction testing to determine the presence of an interaction effect. Concurrently, the p-value corresponding to the interaction was computed for statistical examination to investigate the potential moderating impacts of TyG on the CVD risk. Finally, to explore whether there was a potential non-linear relationship between TyG index and the risk of CVD, several restricted cubic spline curve (RCS) regression models were constructed based on the complete adjustment model, and four knots were placed at the 5th, 35th, 65th, and 95th percentiles. In addition, though the same method, the sex-stratified curves separately were graphed to further explore whether sex differences would have an impact on the predictive value of TyG. Linear regression models were employed to assess the association between the Tyg index and the 10 - year CVD risk, as well as to adjust for potential confounding factors. Sensitivity analyses were conducted, adjusting for eGFR<30mL/1.73m^2^/min, to assess the stability of the results with adjustments for all variables. Mediation analyses were used to investigate whether the relevance of TyG to cardiovascular disease could be explained by glycated hemoglobin after adjusting for factors in Model 4.

The above analyses were performed using SPSS 26.0 and R 4.4.0. Two-tailed P<0.05 was considered statistically significant.

## Results

### Baseline characteristics of study participants

As is shown in **Table 1**, a total of 2987 subjects were included in this study with a mean age of 70.17 ± 6.78 years. Among them, 52.2% were male and 47.8% were female, with an overall prevalence of 32.7%. Compared with the non-CVD population, the CVD population had a higher percentage of males, smokers, hypertensive, and taking antihypertensive drugs (P < 0.05). Race, education level, and marital status were statistically significant differences (P < 0.05). In terms of laboratory indicators, CVD patients had higher levels of FBG, TG, TC, LDL-C, and uric acid, and lower levels of HDL-C, albumin, and eGFR (P < 0.05). Notably, the CVD group exhibited a significantly higher TyG index than the non-CVD group (9.01 vs 8.94, P < 0.05).

**Table 1:** Baseline characteristics of the CVD and non-CVD groups.

Variable	Overall	CVD	Non-CVD	P value
N(%)	2987	977(32.7)	2010(67.3)	
Age, years	70.17(6.78)	71.69(6.70)	69.43(6.70)	<0.001
Sex, n(%)				<0.001
Male	1560(52.2)	560(57.3)	1000(49.8)	
Female	1427(47.8)	417(42.7)	1010(50.2)	
Race, n(%)				<0.001
Mexican American	505(16.9)	129(13.2)	376(18.7)	
Non-Hispanic Black	699(23.4)	204(20.9)	495(24.6)	
Non-Hispanic White	1185(39.7)	482(49.3)	703(35.0)	
Other Hispanic	349(11.7)	98(10.0)	251(12.5)	
Other Race	249(8.3)	64(6.6)	185(9.2)	
Educational level, n(%)				0.018
Less than high school	672(22.5)	206(21.1)	466(23.2)	
High school or equivalent	1206(40.4)	430(44.0)	776(38.6)	
Some college or above	1109(37.1)	341(34.9)	768(38.2)	
Marital status, n(%)				0.003
Married	1633(54.7)	501(51.3)	1132(56.3)	
Widowed	687(23.0)	260(26.6)	427(21.2)	
Divorced	361(12.1)	127(13.0)	234(11.6)	
Never married	306(10.2)	89(9.1)	217(10.8)	
Smoking status, n(%)	1566(35.4)	580(42.0)	986(32.3)	<0.001
Alcohol consumption, n(%)	1703(57.0)	575(58.9)	1128(56.1)	0.214
Hypertension, n(%)				<0.001
Yes	2346(78.5)	820(83.9)	1526(75.9)	
No	641(21.5)	157(16.1)	484(24.1)	
Antihypertensive drugs, n(%)				<0.001
Yes	2134(71.4)	761(77.9)	1373(68.3)	
No	853(28.6)	216(22.1)	637(31.7)	
CKD, n(%)				<0.001
Yes	909(30.4)	399(40.8)	510(25.4)	
No	2078(69.6)	578(59.2)	1500(74.6)	
SBP, mmHg	133.97(32.12)	132.55(33.94)	134.66(31.18)	0.102
DBP, mmHg	65.78(18.35)	63.68(19.00)	66.80(17.94)	<0.001
BMI, kg/m^2^	30.18(8.15)	30.30(9.03)	30.11(7.69)	0.575
FBG, mmol/L	7.15(2.43)	7.30(2.51)	7.08(2.39)	0.022
HbAlc, %	6.99(1.51)	7.06(1.53)	6.96(1.49)	0.102
TG, mmol/L	7.36(5.50,8.94)	7.50(5.65,9.18)	7.28(5.40,8.89)	0.019
TC, mmol/L	4.65(1.11)	4.40(1.14)	4.77(1.08)	<0.001
HDL-C, mmol/L	1.28(0.39)	1.22(0.37)	1.31(0.39)	<0.001
LDL-C, mmol/L	2.71(0.69)	2.60(0.72)	2.76(0.67)	<0.001
Albumin, g/L	41.37(3.53)	40.88(3.39)	41.61(3.31)	<0.001
Uric acid, μmol/L	354.02(94.63)	371.17(105.06)	345.69(87.95)	<0.001
eGFR, mL/1.73m^2^/min	76.28(56.74,99.52)	68.08(48.73,92.86)	80.10(60.90,102.27)	<0.001
TyG	8.96(0.57)	9.01(0.58)	8.94(0.56)	0.003
TyG group				0.015
Q1(<8.67)	752(25.2)	227(23.2)	525(26.1)	
Q2(8.67-8.96)	729(24.4)	223(22.8)	506(25.2)	
Q3(8.96-9.20)	767(25.7)	252(25.8)	515(25.6)	
Q4(>9.20)	739(24.7)	275(28.1)	464(23.1)	

Values are number (percentage), or median (25th-75th percentile).

CKD, chronic kidney disease; SBP, systolic blood pressure; DBP, diastolic blood pressure; BMI, body mass index; FBG, fasting blood glucose; HbA1c, glucated hemoglobin; TG, triglyceride; TC, total cholesterol; HDL-C, high density lipoprotein cholesterol; LDL-C, low density lipoprotein cholesterol; eGFR, estimated-glomerular filtration rate.

Furthermore, we undertook in - depth explorations within five discrete subgroups of CVD by implementing a uniform methodological approach. The prevalence rates were as follows: 11.4% for CHF, 14.0% for CHD, 8.1% for angina, 12.8% for myocardial infarction, and 11.1% for stroke. Notably, the TyG index in the CHD group exhibited a significantly higher value compared to that in the non - CHD group (9.05 vs 8.95, P < 0.05). Conversely, no discernible statistical differences were detected among the remaining four groups (**Supplementary Tables S1**-**S5**).

To further investigate the correlation between TyG index and CVD, the baseline table with TyG index was charted as quartile groups (**Table 2**). The risk of CVD prevalence increased as TyG index grew in each group (Q1: 30.2%; Q2: 30.6%; Q3: 32.9%; and Q4: 37.2%). Various factors including age, sex, race, smoking status, CKD, BMI, FBG, HbA1c%, TG, TC, HDL-C, LDL-C, uric acid, and eGFR demonstrated significant differences between the four quartiles of the TyG index (P < 0.05).

**Table 2:** Baseline characteristics according to TyG index quartiles.

Variable	Quartiles of TyG index				P value
	Q1(<8.67)	Q2(8.67-8.96)	Q3(8.96-9.20)	Q4(>9.20)	
N(%)	752(25.2)	729(24.4)	767(25.7)	739(24.7)	
Age, years	70.91(6.87)	70.16(6.79)	69.90(6.62)	69.71(6.79)	0.003
Sex, n(%)					<0.001
Male	354(47.1)	343(47.1)	460(60.0)	403(54.5)	
Female	398(52.9)	386(52.9)	307(40.0)	336(45.5)	
Race, n(%)					<0.001
Mexican American	101(13.4)	125(17.1)	135(17.6)	144(19.5)	
Non-Hispanic Black	262(34.8)	177(24.3)	151(19.7)	109(14.7)	
Non-Hispanic White	251(33.4)	267(36.6)	327(42.6)	340(46.0)	
Other Hispanic	83(11.0)	85(11.7)	86(11.2)	95(12.9)	
Other Race	55(7.3)	75(10.3)	68(8.9)	51(6.9)	
Educational level, n(%)					0.256
Less than high school	159(21.1)	168(23.0)	169(22.0)	176(23.8)	
High school or equivalent	312(41.5)	274(37.6)	305(39.8)	315(42.6)	
Some college or above	281(37.4)	287(39.4)	293(38.2)	248(33.6)	
Marital status, n(%)					0.071
Married	393(52.3)	372(51.0)	450(58.7)	418(56.6)	
Widowed	186(24.7)	193(26.5)	151(19.7)	157(21.2)	
Divorced	91(12.1)	91(12.5)	87(11.3)	92(12.4)	
Never married	82(10.9)	73(10.0)	79(10.3)	72(9.7)	
Smoking status, n(%)	365(31.7)	379(35.1)	406(35.8)	416(39.2)	<0.001
Alcohol consumption, n(%)	432(57.4)	408(56.0)	450(58.7)	413(55.9)	0.465
Hypertension, n(%)					0.207
Yes	608(80.9)	557(76.4)	605(78.9)	576(77.9)	
No	144(19.1)	172(23.6)	162(21.1)	163(22.1)	
Hypotensive drugs, n(%)					0.4
Yes	552(73.4)	506(69.4)	550(71.7)	526(71.2)	
No	200(26.6)	223(30.6)	217(28.3)	213(28.8)	
CKD, n(%)					0.016
Yes	261(34.7)	224(30.7)	214(27.9)	210(28.4)	
No	491(65.3)	505(69.3)	553(72.1)	529(71.6)	
SBP, mmHg	134.13(34.65)	134.01(31.71)	132.17(30.13)	135.65(31.82)	0.218
DBP, mmHg	65.34(19.44)	65.68(18.86)	65.79(17.54)	66.31(17.70)	0.782
BMI, kg/m^2^	29.39(8.02)	29.92(8.22)	30.99(7.95)	30.39(8.35)	0.001
FBG, mmol/L	6.16(1.10)	6.54(1.33)	6.68(1.24)	9.25(3.66)	<0.001
HbAlc, %	6.60(1.21)	6.91(1.34)	7.03(1.41)	7.44(1.87)	<0.001
TG, mmol/L	4.49(3.56,5.24)	6.83(6.20,7.36)	8.42(8.00,9.02)	10.28(8.89,12.78)	<0.001
TC, mmol/L	4.55(1.04)	4.60(1.09)	4.56(1.07)	4.88(1.22)	<0.001
HDL-C, mmol/L	1.63(0.44)	1.32(0.22)	1.10(0.26)	1.08(0.31)	<0.001
LDL-C, mmol/L	2.56(0.67)	2.72(0.59)	2.79(0.48)	2.77(0.93)	<0.001
Albumin, g/L	41.21(3.49)	41.40(3.32)	41.44(3.33)	41.43(3.27)	0.499
Uric acid, μmol/L	342.37(92.85)	352.38(93.61)	362.22(92.86)	358.98(98.13)	<0.001
eGFR, mL/1.73m^2^/min	72.63(51.89,94.19)	75.38(56.04,98.35)	78.17(59.27,101.02)	79.96(58.82,103.19)	<0.001
Cardiovascular disease, n(%)					0.015
Yes	227(30.2)	223(30.6)	252(32.9)	275(37.2)	
No	525(69.8)	506(69.4)	515(67.1)	464(62.8)	

Values are number (percentage), or median (25th-75th percentile).

CKD, chronic kidney disease; SBP, systolic blood pressure; DBP, diastolic blood pressure; BMI, body mass index; FBG, fasting blood glucose; HbA1c, glucated hemoglobin; TG, triglyceride; TC, total cholesterol; HDL-C, high density lipoprotein cholesterol; LDL-C, low density lipoprotein cholesterol; eGFR, estimated-glomerular filtration rate.

### Association between TyG index and the risk of CVD

After performing univariate logistic regression analyses (**Table 3**), the correlation between TyG index and the risk of CVD can be found through multivariate logistic regression models. The results demonstrated that the higher the TyG index, the higher the risk of CVD in both continuous and categorical variables. In Model 1, the OR for CVD increased with TyG index (OR = 1.235, 95% Cl 1.078-1.414, P = 0.002). In Model 2, age and sex were adjusted (OR = 1.291, 95% Cl 1.124-1.484, P < 0.001), and Model 3 gradually adjusted for more covariates (OR = 1.222, 95% Cl 1.019-1.465, P = 0.030), with all results found to be significant. After adjusting for all covariates, the positive correlation between TyG index and CVD remained consistent (OR = 1.222, 95% Cl 1.016-1.469, P = 0.033), indicating that each unit increased in TyG index was associated with a 22.2% increase in the risk of developing CVD. In the perfectly adjusted model, when the TyG index was divided into quartiles, subjects in the highest quartile group had a significant 48.0% increased risk of CVD compared to the lowest one (OR = 1.480, 95% Cl 1.171-1.871, P = 0.001).

**Table 3:** Logistic regression results showing association between the TyG index and CVD.

	Model 1		Model 2		Model 3		Model 4	
	OR (95%CI)	P	OR (95%CI)	P	OR (95%CI)	P	OR (95%CI)	P
TyG continuous	1.235 (1.078-1.414)	0.002	1.291 (1.124-1.484)	<0.001	1.222 (1.019-1.465)	0.030	1.222 (1.016-1.469)	0.033
TyG categories								
Q1	Ref.		Ref.		Ref.		Ref.	
Q2	1.019 (0.817-1.272)	0.866	1.063 (0.848-1.331)	0.597	1.047 (0.831-1.318)	0.698	1.046 (0.827-1.323)	0.705
Q3	1.132 (0.911-1.405)	0.263	1.157 (0.927-1.443)	0.197	1.171 (0.934-1.469)	0.172	1.126 (0.893-1.420)	0.317
Q4	1.371 (1.105-1.701)	0.004	1.445 (1.160-1.801)	0.001	1.540 (1.225-1.937)	<0.001	1.480 (1.171-1.871)	0.001

Model 1: Unadjusted.

Model 2: Adjusted for age, sex.

Model 3: Adjusted for age, sex, race, educational level, marital status, smoking status, DBP.

Model 4: Adjusted for age, sex, race, educational level, marital status, smoking status, DBP, TC, HDL-C, LDL-C, albumin, eGFR, uric acid, Hypertension, Hypotensive drugs, Chronic kidney disease.

### Subgroup analysis

Subgroup analyses and interaction tests were performed for the association of TyG quartile groups with CVD risk in different subgroups (**Table 4**). The results showed that in the BMI ≥ 24 kg/m^2^ subgroup (OR 1.433, 95% Cl 1.110-1.851, P = 0.006), in the eGFR < 90 mL/1.73m^2^/min subgroup (OR 1.543, 95% Cl 1.165-2.042, P = 0.002), and the subgroups without chronic kidney disease (OR 1.505, 95% Cl 1.111 -2.037, P = 0.008), and with hypertension (OR 1.449, 95% Cl 1.122-1.872, P = 0.004), individuals in the highest TyG quartile group presented a higher risk of CVD compared with individuals in the lowest TyG Quartile group. There was a statistically significant interaction among individuals without hypertension in groups Q2 and Q3. Interaction was presented for the BMI subgroup in group Q2 and the CKD subgroup in group Q3, and no significant interaction was found among the remaining subgroups (all P for interaction > 0.05).

**Table 4:** Subgroup analysis for the association between the TyG index and the risk of CVD.

Subgroups	Q1	Q2	P	Q3	P	Q4	P
	OR (95%Cl)	OR (95%Cl)		OR (95%Cl)		OR (95%Cl)	
Sex Male Female P interaction	Ref.	1.288 (0.919-1.803) 0.905 (0.644-1.272)	0.142 0.565 0.790	1.151 (0.832-1.592) 1.164 (0.818-1.656)	0.396 0.399 0.804	1.538 (1.108-2.135) 1.409 (0.998-1.988)	0.010 0.051 0.897
BMI, kg/m^2^ < 24 ≥ 24 P interaction	Ref.	1.441 (0.807-2.573) 1.009 (0.776-1.312)	0.216 0.946 0.035	1.056 (0.535-2.084) 1.129 (0.875-1.458)	0.876 0.350 0.086	1.500 (0.770-2.924) 1.433 (1.110-1.851)	0.234 0.006 0.166
eGFR, mL/1.73m^2^/min < 90 ≥ 90 P interaction	Ref.	1.158 (0.879-1.525) 0.863 (0.535-1.391)	0.299 0.545 0.076	1.160 (0.878-1.531) 1.090 (0.691-1.718)	0.296 0.711 0.588	1.543 (1.165-2.042) 1.372 (0.979-2.146)	0.002 0.165 0.778
CKD Yes No P interaction	Ref.	0.908 (0.622-1.328) 1.181 (0.868-1.607)	0.620 0.289 0.700	1.036 (0.704-1.525) 1.219 (0.900-1.652)	0.856 0.201 0.014	1.407 (0.957-2.069) 1.505 (1.111-2.037)	0.082 0.008 0.733
Hypertension Yes No P interaction	Ref.	0.891 (0.686-1.158) 2.464 (1.350-4.498)	0.389 0.003 0.848	1.035 (0.800-1.339) 1.972 (1.055-3.688)	0.791 0.033 0.875	1.449 (1.122-1.872) 1.853 (0.978-3.510)	0.004 0.058 0.812

Each subgroup analysis was adjusted for age, sex, race, educational level, marital status, smoking status, DBP, TC, HDL-C, LDL-C, albumin, eGFR, uric acid, Hypertension, Hypotensive drugs, Chronic kidney disease.

### RCS analysis

**Figure 2:**
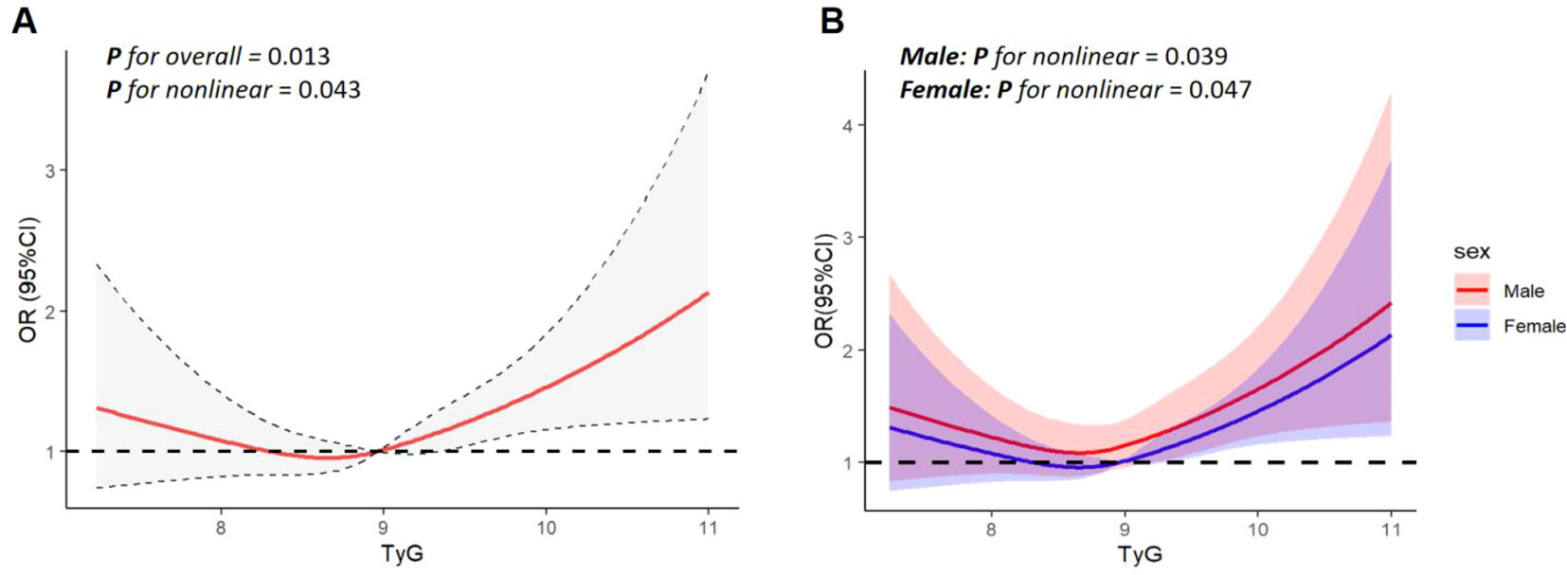
The restricted cubic spline (RCS) analysis of the association between the TyG index and the risk of CVD. **(A)** RCS curve of the association between TyG index and CVD risk in the total population; **(B)** RCS curves of the association between TyG index and CVD risk among males and females. The association was adjusted for age, sex, race, educational level, marital status, smoking status, DBP, TC, HDL-C, LDL-C, albumin, eGFR, uric acid, Hypertension, Hypotensive drugs, Chronic kidney disease.

**Figure 2A** showed a significant nonlinear relationship between the TyG index and CVD risk in the form of a U-shaped curve (with an overall P-value of 0.013, a nonlinear P-value of 0.043, and an inflection point at 8.63). To the left of the inflection point, the TyG index was uncorrelated with CVD risk but positively correlated to the right.

**Figure 2B** indicated that the TyG index was nonlinearly associated with CVD risk in both genders, aligning with the pattern in the total population. The inflection points of TyG levels for men and women were 8.61 (nonlinear P = 0.039) and 8.68 (nonlinear P = 0.047), respectively. There was no association to the left of these points, but a positive link existed on the right.

### Sensitivity analysis

In **Table 5**, the stability of the aforesaid research finding were verified s via sensitivity analysis. Considering that severely impaired kidney function may influence the evaluation of the cardiovascular risk in diabetic patients, subjects with eGFR < 30 mL/1.73m^2^/min were excluded when adjusting confounding factors. Multivariate logistic regression analysis demonstrated that the TyG index was significantly correlated with CVD risk (P < 0.05) both as a continuous and categorical variable in Model 1 and Model 2. In Model 3, a one-unit increase in the TyG index led to a 21.0% rise in CVD risk (OR = 1.210, 95% CI 1.042 - 1.406, P = 0.012). Additionally, the CVD risk in the Q4 group was 1.279 times that of the Q1 group (OR = 1.279, 95% CI 1.010 - 1.619, P = 0.041). In the fully adjusted Model 4, a higher TyG index was still significantly associated with a higher CVD risk (continuous variable: OR = 1.222, 95% CI 1.008 - 1.480, P = 0.041; Q4 vs Q1: OR = 1.436, 95% CI 1.124 - 1.835, P = 0.004), emphasizing the high credibility and robustness of results.

**Table 5:** Sensitivity analysis.

	Model 1		Model 2		Model 3		Model 4	
	OR (95%CI)	P	OR (95%CI)	P	OR (95%CI)	P	OR (95%CI)	P
TyG continuous	1.209 (1.050-1.392)	0.008	1.258 (1.088-1.455)	0.002	1.210 (1.042-1.406)	0.012	1.222 (1.008-1.480)	0.041
TyG categories								
Q1	Ref.		Ref.		Ref.		Ref.	
Q2	0.955 (0.758-1.203)	0.696	0.994 (0.786-1.257)	0.959	0.970 (0.764-1.232)	0.806	1.011 (0.789-1.295)	0.932
Q3	1.087 (0.869-1.361)	0.463	1.094 (0.870-1.376)	0.442	1.061 (0.839-1.340)	0.622	1.099 (0.861-1.404)	0.448
Q4	1.315 (1.052-1.645)	0.016	1.377 (1.095-1.730)	0.006	1.279 (1.010-1.619)	0.041	1.436 (1.124-1.835)	0.004

Excluded subjects with eGFR < 30 mL/1.73m^2^/min.

Model 1: Unadjusted.

Model 2: Adjusted for age, sex.

Model 3: Adjusted for age, sex, race, educational level, marital status, smoking status, DBP.

Model 4: Adjusted for age, sex, race, educational level, marital status, smoking status, DBP, TC, HDL-C, LDL-C, albumin, eGFR, uric acid, Hypertension, Hypotensive drugs, Chronic kidney disease.

### The association of the TyG index with 10 - year risk of CVD

**Table 6** demonstrated the associations between the TyG index and 10 - year CVD risk via four distinct linear regression models, incrementally adjusting potential confounding factors. Remarkably, a consistently significant positive correlation emerged. Across all models, the data clearly showed that as the TyG index rose, the 10 - year CVD risk increased substantially. In the fully adjusted Model 4, the corresponding regression coefficients of TyG index were 0.228 (95%CI 0.098 - 0.357, P = 0.001) for 10 - year CVD risk.

**Table 6:** Linear regression models of associations between the TyG index and 10-year risk of CVD.

10-year risk of CVD	Model 1		Model 2		Model 3		Model 4	
	β (95%CI)	P	β (95%CI)	P	β (95%CI)	P	β (95%CI)	P
	0.241 (0.020-0.461)	0.033	0.335 (0.148-0.521)	<0.001	0.286 (0.138-0.434)	<0.001	0.228 (0.098-0.357)	0.001

Model 1: Unadjusted.

Model 2: Adjusted for age, sex.

Model 3: Adjusted for age, sex, race, educational level, marital status, smoking status, DBP.

Model 4: Adjusted for age, sex, race, educational level, marital status, smoking status, DBP, TC, HDL-C, LDL-C, albumin, eGFR, uric acid, Hypertension, Hypotensive drugs, Chronic kidney disease.

### Mediation analysis of TyG with CVD risk

Mediation analysis showed that HbA1c mediated the correlation between TyG and the risk of CVD. The percentage of the indirect effect of the correlation between TyG and CVD risk mediated by HbA1c was 12.5% (**Supplementary Figure S1**).

## Discussion

Here, the relationship between TyG index and the risk of CVD in 2987 diabetic patients aged 60 years and above was investigated based on information from the NAHANES database from the period 2007 to 2016. Excitingly, there is a significant nonlinear relationship between the TyG index and CVD, and there seemingly exists a threshold effect. This reveals that both overly high and low TyG values will elevate the CVD risk. Moreover, this study has verified that a pronounced U-shaped association between the TyG index and CVD risk prevails in both genders. The inflection points have been pinpointed as 8.61 for men and 8.68 for women. This implies that in clinical practice, the TyG index can function as a crucial predictor for gauging the CVD occurrence risk in the elderly diabetic population and facilitate the formulation of targeted CVD prevention and personalized treatment strategies.

The TyG index, a new indicator for evaluating insulin resistance, is widely applied in multiple fields, such as the screening of the general population, the management of diabetic patients, and the monitoring of cardiovascular diseases. A longitudinal cohort study showed that the TyG index could serve as a useful indicator for predicting the glycemic conversion outcome in people with prediabetes. This study demonstrated a negative, non - linear link between the TyG index and glucose status conversion from prediabetes to normoglycemia (14). Gao et al., through a cross-sectional study, were the first to discover a positive correlation between the TyG index and impaired cardiovascular fitness among 3,364 non-diabetic young people (15). The study by Zhang et al. observed a positive correlation between the TyG index and both CVD mortality and all - cause mortality in CVD patients with diabetes or pre - diabetes. Specifically, when the TyG index exceeds certain thresholds (8.5 for CVD mortality and 8.2 for all - cause mortality), the risk of death increases significantly (16). A study conducted in Italy showed that, among elderly hypertensive individuals with prediabetes, the TyG index served as a valuable reference for predicting the risks of both cognitive and physical impairments, and even frailty (17). Revealed by Zhao et al.’s research, the TyG index exhibited a positive correlation with the incidence of chest pain and was capable of predicting the all-cause mortality of patients suffering from chest pain (18). Wu et al.’s longitudinal survey of 4,710 middle-aged and elderly people aged over 45 showed that participants with a higher baseline TyG level had a higher incidence of stroke (19). As was demonstrated, the TyG index served as an important predictor and therapeutic target for heart failure (HF) as well, with a positive correlation to the risk of HF (20). In addition, it was indicated by Xue et al.’s research that high clinical value was also possessed by TyG-related parameters in identifying liver diseases closely associated with metabolic disorders, such as non-alcoholic fatty liver disease (NAFLD) and metabolic associated fatty liver disease (MAFLD) (21).

More significantly, the nonlinear association between the TyG index and CVD risk has currently been explored across various populations. A prospective cohort study involving 7,851 subjects observed a U-shaped association between the TyG index and cardiovascular mortality in the general population, with a threshold of 8.7. After exceeding this threshold, a positive correlation between the TyG index and CVD risk was only detected in male subjects (22). Mengjie Zhao et al. also identified this particular U-shaped association among middle-aged and elderly diabetic patients aged 45 and above and concluded that there was a significant correlation between TyG and CVD mortality in men at the TyG > 9 (23). Sangsang Li et al.’s retrospective analysis of 6,078 subjects aged 60 and above also suggested a nonlinear relationship between the TyG index and CVD risk. Conversely, they found that when the TyG value was greater than the threshold (9.53), TyG was a stronger risk factor for CVD in women compared to men (24). Generally, a higher TyG index indicates higher insulin resistance in the body and can trigger a series of pathophysiological reactions such as elevated blood glucose, abnormal increase in triglycerides, and enhanced inflammatory response, thus substantially increasing the risk of CVD (25–27). Meanwhile, individuals with a low TyG level may have an increased CVD risk due to reasons like sympathetic nerve stimulation caused by hypoglycemia (28, 32). However, there is no definite mechanism to explain how gender differences regulate the impact of TyG on CVD risk. Moreover, the results are inconsistent due to variations in sample size, follow-up duration, and target populations. It has been reported that it may be related to the protective effect of sex steroid hormones, especially estrogen, in women (30). Therefore, it is of significant clinical importance to explore the impact of the TyG index on CVD risk and determine its threshold, especially in the elderly diabetic population, as it can help reduce the occurrence risk of CVD to some extent.

Furthermore, according to our subgroup analysis, this study reveals the correlations of the TyG index among the populations with cardiovascular comorbidities or other pathologies, including overweight, renal impairment, and the presence or absence of CKD or hypertension. The strong associations remain even after adjusting for confounding factors, suggesting that the findings are applicable to most people. It emphasizes that the TyG index can serve as a valuable marker for assessing cardiovascular risks in diverse populations, consistent with other studies. A longitudinal study by Xin et al. with 15,056 physical examinees as samples and a 12-year follow-up found that the trajectories of participants whose TyG indices showed “moderate increasing” and “high stable” were closely related to the risk of hypertension (31). A cohort study of 6,114 Chinese aged 45 years or older by Ye Zixiang et al. showed that increased TyG index enhanced the association between diabetes and CVD in middle-aged and older adults (32). Their subgroup analysis found that subjects with concomitant hypertension had an increased CVD risk, consistent with the results of this study. Delightfully, Wanlu Su precisely confirmed this in a prospective observational study of 4,434 people with diabetes complicated by hypertension, stating that those with higher TyG index levels were more likely to have increased incidences of T2DM-HTN comorbidities (33). This may imply that the metabolic pathways of hypertension and the TyG index interact with each other and cause synergistic damage to the cardiovascular system (28, 29). Mengjie Zhao et al. noted a significant positive correlation between the TyG index and CVD risk in the subgroup with BMI ≥ 25 kg/m^2^, which is also concluded in this study (34). This fully demonstrates that high BMI may lead to increased insulin resistance and abnormal fat metabolism, thus affecting the TyG level. Conversely, a high TyG index indicates abnormal glucose and lipid metabolism in the body, prompting abnormal fat accumulation and resulting in high BMI. The interaction between the two significantly increases an individual’s risk of CVD. Cancan Cui et al. illustrated in a prospective cohort study of 6496 subjects, through stratified analysis, that in diabetic patients, the combination of a higher TyG index and a lower eGFR level (eGFR < 60 mL/1.73m^2^/min) was associated with the risk of cardiovascular disease, consistent with this study (34). Therefore, it can be speculated that renal impairment can significantly mediate the association between the TyG index and cardiovascular risk. This may be related to the renal metabolic feedback mechanism. In CKD patients, the kidney’s clearance ability for substances decreases, leading to elevated levels of triglycerides and glucose in the blood. Meanwhile, persistently high TyG index may lead to renal microangiopathy, which further impairs renal function (35). In summary, understanding the interactions between TyG and hypertension, BMI, and CKD helps to stratify patients’ risks more precisely, comprehensively assess the risks of adverse events such as cardiovascular diseases and kidney disease progression, and provide effective treatment strategies to control the conditions, thereby alleviating the progression of kidney diseases and preventing cardiovascular complications (36–39). This also means that the TyG index can be a powerful predictor of the risk of cardiovascular diseases in elderly diabetic patients.

Although most studies and ours have consistently confirmed the correlation between TyG and CVD, the mechanisms involved require further discussion. Presently, the published papers unanimously agree on the following mechanisms: (1) The body’s sensitivity to insulin is reduced, causing chronic hyperglycemia, which induces chronic fibrosis of myocardial tissue and myocardial remodeling, leading to the development of cardiovascular disease (32); (2) Hyperinsulinemia makes fat ineffectively used, causing dyslipidemia (33), exacerbating inflammatory responses and oxidative stress, producing lipotoxicity in the heart, damaging endothelial cells and vascular function (34), and even causing heart failure (34); (3) At the same time, the inflammatory response will increase platelet aggregation, promote thrombosis and hypercoagulable state of the body (35), cause atherosclerotic dyslipidemia (36), and further deterioration of cardiovascular disease; (4) Elevated insulin levels help to excite the sympathetic nervous system, resulting in increased adrenaline secretion, higher blood pressure, renal sodium storage, and increased cardiac load, leading to cardiovascular and renal damage (37, 38); (5) Furthermore, insulin resistance may promote the development and progression of cardiovascular diseases through subclinical conditions (39); (6) Favorable insulin sensitivity is conducive to the maintenance of normal glycometabolism, attenuates the adverse effects of hyperglycemia on the cardiovascular system, confers a protective function on the cardiovascular apparatus, and mitigates myocardial damage (34, 40); and (7) Potential confounding factors not included in the study may also influence the relationship between TyG and CVD risk. Individuals who follow a high-sugar and high-fat diet for a long time are prone to obesity and abnormal blood glucose and lipid levels, directly elevating the TyG index (41, 42). Regular exercise can improve insulin sensitivity, reduce the TyG index, and simultaneously enhance cardiovascular function and lower CVD risk (43). Constraints like financial difficulties can lead to inadequate preventive care and may also affect the CVD risk in the diabetic population (44).

Despite the meaningful findings of our study, some limitations remain unavoidable: (1) This is a retrospective observational study without prospective design and interventions, thus the causal association between TyG and CVD cannot be confirmed, which needs further exploration; (2) The data in this study were obtained from NHANES Epidemiological Survey, and the subjects were generally healthy community population, which was different from clinical data, and most of them may have HDL-C in normal range. Therefore, further validation of hospital cohort data is still needed before clinical application; (3) As mentioned above, NHANES, as a multiple sampling design, needs to be reweighted if it is intended to be representative of the U.S. population as a whole. However, in our study, we did not perform any data adjustments because we were analyzing in deeper subgroups and adjusting the weights could have missed some important effects, which also means that our findings may not be sustainable for the entire U.S. population; (4) Although this study included as many variables as it could and adjustment for confounders was made wherever possible in the regression analyses, potential confounders, such as genetic susceptibility, genetic variation, dietary and environmental factors and mental health and social factors, were still unavoidable; (5) Finally, the diagnoses of diabetes and CVD partly relied on NHANES participants’ recall during interviews, causing recall bias. When assessing the TyG-CVD relationship, patients may misremember onset time, treatment, or lifestyle changes, distorting the true link. Individual differences in memory and attention levels are large. High-stress individuals often recall inaccurately due to overlooking details, making the results uncertain; (6) The cross-sectional nature of NHANES data may affect the results. It is unclear whether TyG fluctuations precede or follow the latent changes of CVD, making it difficult to prove causality. Also, it is hard to capture the short-term impacts of seasons and sudden incidents. Geographically, metabolic fluctuation patterns vary among populations in regions with different climates and social rhythms. Thus, the results cannot align with the actual TyG-CVD dynamics everywhere, weakening their generalizability; and (7) The lack of external validation is a key constraint. Future studies need to replicate these findings in independent cohorts to confirm the robustness of the conclusions.

## Conclusions

There is an independent correlation between TyG index and cardiovascular disease in elderly diabetic subjects. TyG index can be introduced into the routine screening and management of elderly diabetic patients for early detection, diagnosis, and treatment. In clinical practice, physicians can stratify the risk of CVD for elderly diabetic patients based on the relationship between the TyG index and CVD. For patients whose TyG index is close to or exceeds the inflection point value, the risk of CVD occurrence should be monitored more closely, and proactive intervention measures should be taken. Additionally, it is advisable to combine the TyG index with other CVD risk factors such as age, blood pressure, blood lipids, blood glucose control status, and lifestyle to construct a more accurate CVD risk prediction model. This will enhance the predictive ability for CVD occurrence in the elderly diabetic population, thereby quickly and effectively reducing the impact of the TyG index on CVD and decreasing the morbidity and mortality of CVD.

## Data Availability

The original contributions presented in the study are included in the article/**Supplementary Material**, further inquiries can be directed to the corresponding author/s.

## Supplementary material

Supplementary Table 1 Baseline characteristics of the CHF and non-CHF groups.

Supplementary Table 2 Baseline characteristics of the CHD and non-CHD groups.

Supplementary Table 3 Baseline characteristics of the Angina and non-Angina groups.

Supplementary Table 4 Baseline characteristics of the myocardial infarction and non-myocardial infarction groups.

Supplementary Table 5 Baseline characteristics of the Stroke and non-Stroke groups.

Supplementary Figure 1 Mediation analysis of TyG with CVD risk. Adjusted for age, sex, race, educational level, marital status, smoking status, DBP, TC, HDL-C, LDL-C, albumin, eGFR, uric acid, Hypertension, Hypotensive drugs, Chronic kidney disease.
