# Supplementary material for: The triglyceride–glucose index is a promising predictor for the risk of cardiovascular disease in the diabetic population aged ≥60 years in the United States: a retrospective cohort study from NHANES (2007-2016)
Source: Front Endocrinol (Lausanne). 2025 Feb 21;16:1475590. doi: 10.3389/fendo.2025.1475590 (PMC11885141; doi:10.3389/fendo.2025.1475590)
Supplement: Supplementary Table 1 — Baseline characteristics of the CHF and non-CHF groups. [file DataSheet1.docx]

Suppl. Table 1 Baseline characteristics of the CHF and non-CHF groups

| Variable | CHF | Non-CHF | P value |
| --- | --- | --- | --- |
| N(%) | 341(11.4) | 2646(88.6) |  |
| Age, years | 71.73(6.73) | 69.97(6.76) | <0.001 |
| Sex, n(%)  Male  Female | 185(54.3)  156(45.7) | 1375(52.0)  1271(48.0) | 0.426 |
| Race, n(%)  Mexican American  Non-Hispanic Black  Non-Hispanic White  Other Hispanic  Other Race | 38(11.1)  35(10.3)  172(50.4)  81(23.8)  15(4.4) | 467(17.6)  314(11.9)  1013(38.3)  618(23.4)  234(8.8) | <0.001 |
| Educational level, n(%)  Less than high school  High school or equivalent  Some college or above | 75(22.0)  147(43.1)  119(34.9) | 597(22.6)  1059(40.0)  990(37.4) | 0.530 |
| Marital status, n(%)  Married  Widowed  Divorced  Never married | 157(46.0)  110(32.3)  43(12.6)  31(9.1) | 1476(55.8)  577(21.8)  318(12.0)  275(10.4) | <0.001 |
| Smoking status, n(%) | 198(58.1) | 1368(51.7) | 0.038 |
| Alcohol consumption, n(%) | 194(56.9) | 1509(57.0) | 0.649 |
| Hypertension, n(%)  Yes  No | 297(87.1)  44(12.9) | 2049(77.4)  597(22.6) | <0.001 |
| Antihypertensive drugs, n(%)  Yes  No | 283(83.0)  58(17.0) | 1851(70.0)  795(30.0) | <0.001 |
| CKD, n(%)  Yes  No | 172(50.4)  169(49.6) | 737(27.9)  1909(72.1) | <0.001 |
| SBP, mmHg | 127.79(38.46) | 134.77(31.13) | 0.001 |
| DBP, mmHg | 60.89(20.93) | 66.41(17.89) | <0.001 |
| BMI, kg/m^2^ | 30.74(10.27) | 30.10(7.84) | 0.268 |
| FBG, mmol/L | 7.15(2.42) | 7.15(2.44) | 0.939 |
| HbAlc, % | 7.03(1.56) | 6.99(1.50) | 0.616 |
| TG, mmol/L | 7.76(5.64, 9.33) | 7.36(5.50, 8.92) | 0.098 |
| TC, mmol/L | 4.37(1.17) | 4.68(1.10) | <0.001 |
| HDL-C, mmol/L | 1.20(0.36) | 1.29(0.39) | <0.001 |
| LDL-C, mmol/L | 2.64(0.76) | 2.72(0.68) | 0.065 |
| Albumin, g/L | 40.22(3.63) | 41.52(3.29) | <0.001 |
| Uric acid, μmol/L | 393.55(121.39) | 348.93(89.38) | <0.001 |
| eGFR, mL/1.73m^2^/min | 62.46(41.55, 88.98) | 77.59(58.92, 100.31) | <0.001 |
| TyG | 8.99(0.57) | 8.96(0.57) | 0.263 |
| TyG group  Q1(<8.67)  Q2(8.67-8.96)  Q3(8.96-9.20)  Q4(>9.20) | 89(26.1)  67(19.6)  95(27.9)  90(26.4) | 663(25.1)  662(25.0)  672(25.4)  649(24.5) | 0.184 |

Suppl. Table 2 Baseline characteristics of the CHD and non-CHD groups

| Variable | CHD | Non-CHD | P value |
| --- | --- | --- | --- |
| N(%) | 417(14.0) | 2570(86.0) |  |
| Age, years | 71.57(6.66) | 69.94(6.77) | <0.001 |
| Sex, n(%)  Male  Female | 280(67.1)  137(32.9) | 1280(49.8)  1290(50.2) | <0.001 |
| Race, n(%)  Mexican American  Non-Hispanic Black  Non-Hispanic White  Other Hispanic  Other Race | 58(13.9)  36(8.6)  234(56.1)  53(12.7)  36(8.6) | 447(17.4)  313(12.2)  951(37.0)  646(25.1)  213(8.3) | <0.001 |
| Educational level, n(%)  Less than high school  High school or equivalent  Some college or above | 80(19.2)  174(41.7)  163(39.1) | 592(23.0)  1032(40.2)  946(36.8) | 0.214 |
| Marital status, n(%)  Married  Widowed  Divorced  Never married | 239(57.3)  104(24.9)  46(11.0)  28(6.7) | 1394(54.2)  583(22.7)  315(12.3)  278(10.8) | 0.051 |
| Smoking status, n(%) | 258(61.9) | 1308(50.9) | <0.001 |
| Alcohol consumption, n(%) | 255(61.2) | 1448(56.3) | 0.157 |
| Hypertension, n(%)  Yes  No | 338(81.1)  79(18.9) | 2008(78.1)  562(21.9) | 0.177 |
| Antihypertensive drugs, n(%)  Yes  No | 317(76.0)  100(24.0) | 1817(70.7)  753(29.3) | 0.026 |
| CKD, n(%)  Yes  No | 163(39.1)  254(60.9) | 746(29.0)  1824(71.0) | <0.001 |
| SBP, mmHg | 131.94(34.03) | 134.30(31.80) | 0.164 |
| DBP, mmHg | 63.45(19.07) | 66.15(18.20) | 0.005 |
| BMI, kg/m^2^ | 30.14(8.29) | 30.18(8.13) | 0.929 |
| FBG, mmol/L | 7.43(2.60) | 7.10(2.40) | 0.017 |
| HbAlc, % | 7.12(1.43) | 6.97(1.52) | 0.066 |
| TG, mmol/L | 7.76(5.78, 9.33) | 7.36(5.40, 8.92) | 0.008 |
| TC, mmol/L | 4.27(1.06) | 4.71(1.11) | <0.001 |
| HDL-C, mmol/L | 1.19(0.33) | 1.30(0.40) | <0.001 |
| LDL-C, mmol/L | 2.52(0.69) | 2.74(0.69) | <0.001 |
| Albumin, g/L | 41.25(3.10) | 41.39(3.39) | 0.403 |
| Uric acid, μmol/L | 372.67(98.83) | 351.00(93.60) | <0.001 |
| eGFR, mL/1.73m^2^/min | 68.34(49.53, 94.23) | 77.51(57.65, 100.29) | <0.001 |
| TyG | 9.05(0.56) | 8.95(0.57) | 0.001 |
| TyG group  Q1(<8.67)  Q2(8.67-8.96)  Q3(8.96-9.20)  Q4(>9.20) | 88(21.1)  94(22.5)  111(26.6)  124(29.7) | 664(25.8)  635(24.7)  656(25.5)  615(23.9) | 0.030 |

Suppl. Table 3 Baseline characteristics of the Angina and non-Angina groups

| Variable | Angina | Non-Angina | P value |
| --- | --- | --- | --- |
| N(%) | 242(8.1) | 2745(91.9) |  |
| Age, years | 70.41(6.98) | 70.15(6.76) | 0.562 |
| Sex, n(%)  Male  Female | 140(57.9)  102(42.1) | 1420(51.7)  1325(48.3) | 0.068 |
| Race, n(%)  Mexican American  Non-Hispanic Black  Non-Hispanic White  Other Hispanic  Other Race | 38(15.7)  28(11.6)  127(52.5)  34(14.0)  15(6.2) | 467(17.0)  321(11.7)  1058(38.5)  665(24.2)  234(8.5) | <0.001 |
| Educational level, n(%)  Less than high school  High school or equivalent  Some college or above | 48(19.8)  104(43.0)  90(37.2) | 624(22.7)  1102(40.1)  1019(37.1) | 0.530 |
| Marital status, n(%)  Married  Widowed  Divorced  Never married | 132(54.5)  58(24.0)  38(15.7)  14(5.8) | 1501(54.7)  629(22.9)  323(11.8)  292(10.6) | 0.045 |
| Smoking status, n(%) | 146(60.3) | 1420(51.7) | 0.034 |
| Alcohol consumption, n(%) | 142(58.7) | 1561(56.9) | 0.820 |
| Hypertension, n(%)  Yes  No | 206(85.1)  36(14.9) | 2140(78.0)  605(22.0) | 0.009 |
| Antihypertensive drugs, n(%)  Yes  No | 197(81.4)  45(18.6) | 1936(70.6)  808(29.4) | <0.001 |
| CKD, n(%)  Yes  No | 88(36.4)  154(63.6) | 821(29.9)  1924(70.1) | 0.036 |
| SBP, mmHg | 131.18(30.01) | 134.22(32.29) | 0.159 |
| DBP, mmHg | 64.34(18.33) | 65.90(18.35) | 0.203 |
| BMI, kg/m^2^ | 30.77(9.01) | 30.13(8.07) | 0.242 |
| FBG, mmol/L | 7.34(2.60) | 7.13(2.42) | 0.195 |
| HbAlc, % | 7.00(1.25) | 6.99(1.53) | 0.981 |
| TG, mmol/L | 7.64(5.76, 9.17) | 7.36(5.50, 8.92) | 0.216 |
| TC, mmol/L | 4.42(1.13) | 4.67(1.11) | 0.001 |
| HDL-C, mmol/L | 1.19(0.32) | 1.29(0.39) | <0.001 |
| LDL-C, mmol/L | 2.62(0.69) | 2.72(0.69) | 0.036 |
| Albumin, g/L | 41.11(3.12) | 41.39(3.37) | 0.206 |
| Uric acid, μmol/L | 374.38(101.03) | 352.23(93.85) | 0.001 |
| eGFR, mL/1.73m^2^/min | 71.82(50.40, 97.07) | 76.68(57.09, 99.60) | 0.111 |
| TyG | 9.02(0.55) | 8.96(0.57) | 0.081 |
| TyG group  Q1(<8.67)  Q2(8.67-8.96)  Q3(8.96-9.20)  Q4(>9.20) | 50(20.7)  58(24.0)  64(26.4)  70(28.9) | 702(25.6)  671(24.4)  703(25.6)  669(24.4) | 0.253 |

Suppl. Table 4 Baseline characteristics of the myocardial infarction and non-myocardial infarction groups

| Variable | Myocardial infarction | Non-myocardial infarction | P value |
| --- | --- | --- | --- |
| N(%) | 383(12.8) | 2604(87.2) |  |
| Age, years | 71.38(6.57) | 69.99(6.79) | <0.001 |
| Sex, n(%)  Male  Female | 251(65.5)  132(34.5) | 1309(50.3)  1295(49.7) | <0.001 |
| Race, n(%)  Mexican American  Non-Hispanic Black  Non-Hispanic White  Other Hispanic  Other Race | 50(13.1)  46(12.0)  199(52.0)  65(17.0)  23(6.0) | 455(17.5)  303(11.6)  986(37.9)  634(24.3)  226(8.7) | <0.001 |
| Educational level, n(%)  Less than high school  High school or equivalent  Some college or above | 86(22.5)  168(43.9)  129(33.7) | 586(22.5)  1038(39.9)  980(37.6) | 0.255 |
| Marital status, n(%)  Married  Widowed  Divorced  Never married | 205(53.5)  95(24.8)  49(12.8)  34(8.9) | 1428(54.8)  592(22.7)  312(12.0)  272(10.4) | 0.634 |
| Smoking status, n(%) | 251(65.5) | 1315(50.5) | <0.001 |
| Alcohol consumption, n(%) | 239(62.4) | 1464(56.2) | 0.071 |
| Hypertension, n(%)  Yes  No | 314(82.0)  69(18.0) | 2032(78.0)  572(22.0) | 0.079 |
| Antihypertensive drugs, n(%)  Yes  No | 293(76.5)  90(23.5) | 1841(70.7)  763(29.3) | 0.019 |
| CKD, n(%)  Yes  No | 161(42.0)  222(58.0) | 748(28.7)  1856(71.3) | <0.001 |
| SBP, mmHg | 131.60(36.21) | 134.32(31.49) | 0.163 |
| DBP, mmHg | 63.71(18.74) | 66.08(18.27) | 0.018 |
| BMI, kg/m^2^ | 30.02(8.38) | 30.20(8.12) | 0.688 |
| FBG, mmol/L | 7.27(2.63) | 7.13(2.41) | 0.285 |
| HbAlc, % | 7.05(1.39) | 6.99(1.52) | 0.444 |
| TG, mmol/L | 7.46(5.61, 9.33) | 7.36(5.50, 8.92) | 0.316 |
| TC, mmol/L | 4.34(1.06) | 4.69(1.11) | <0.001 |
| HDL-C, mmol/L | 1.18(0.33) | 1.30(0.40) | <0.001 |
| LDL-C, mmol/L | 2.57(0.70) | 2.73(0.69) | <0.001 |
| Albumin, g/L | 40.69(3.38) | 41.47(3.34) | <0.001 |
| Uric acid, μmol/L | 375.52(108.94) | 350.86(91.94) | <0.001 |
| eGFR, mL/1.73m^2^/min | 68.57(49.47, 90.68) | 77.52(57.66, 100.46) | <0.001 |
| TyG | 9.00(0.59) | 8.96(0.56) | 0.151 |
| TyG group  Q1(<8.67)  Q2(8.67-8.96)  Q3(8.96-9.20)  Q4(>9.20) | 96(25.1)  90(23.5)  85(22.2)  112(29.2) | 656(25.2)  639(24.5)  682(26.2)  627(24.1) | 0.120 |

Suppl. Table 5 Baseline characteristics of the Stroke and non-Stroke groups

| Variable | Stroke | Non-Stroke | P value |
| --- | --- | --- | --- |
| N(%) | 332(11.1) | 2655(88.9) |  |
| Age, years | 72.31(6.69) | 69.90(6.74) | <0.001 |
| Sex, n(%)  Male  Female | 175(52.7)  157(47.3) | 1385(52.2)  1270(47.8) | 0.851 |
| Race, n(%)  Mexican American  Non-Hispanic Black  Non-Hispanic White  Other Hispanic  Other Race | 46(13.9)  25(7.5)  155(46.7)  81(24.4)  25(7.5) | 459(17.3)  324(12.2)  1030(38.8)  618(23.3)  224(8.4) | 0.013 |
| Educational level, n(%)  Less than high school  High school or equivalent  Some college or above | 69(20.8)  153(46.1)  110(33.1) | 603(22.7)  1053(39.7)  999(37.6) | 0.078 |
| Marital status, n(%)  Married  Widowed  Divorced  Never married | 165(49.7)  91(27.4)  47(14.2)  29(8.7) | 1468(55.3)  596(22.4)  314(11.8)  277(10.4) | 0.072 |
| Smoking status, n(%) | 189(56.9) | 1377(51.9) | 0.188 |
| Alcohol consumption, n(%) | 184(55.4) | 1519(57.2) | 0.310 |
| Hypertension, n(%)  Yes  No | 287(86.4)  45(13.6) | 2059(77.6)  596(22.4) | <0.001 |
| Antihypertensive drugs, n(%)  Yes  No | 266(80.1)  66(19.9) | 1868(70.4)  787(29.6) | <0.001 |
| CKD, n(%)  Yes  No | 148(44.6)  184(55.4) | 761(28.7)  1894(71.3) | <0.001 |
| SBP, mmHg | 131.93(36.80) | 134.23(31.49) | 0.276 |
| DBP, mmHg | 63.58(19.74) | 66.18(18.13) | 0.001 |
| BMI, kg/m^2^ | 29.09(9.70) | 30.31(7.93) | 0.027 |
| FBG, mmol/L | 7.21(2.47) | 7.14(2.43) | 0.627 |
| HbAlc, % | 7.01(1.63) | 6.99(1.49) | 0.847 |
| TG, mmol/L | 7.46(5.29, 9.02) | 7.36(5.55, 8.94) | 0.957 |
| TC, mmol/L | 4.48(1.21) | 4.67(1.10) | 0.005 |
| HDL-C, mmol/L | 1.25(0.43) | 1.29(0.38) | 0.169 |
| LDL-C, mmol/L | 2.64(0.75) | 2.72(0.68) | 0.088 |
| Albumin, g/L | 40.67(3.42) | 41.46(3.33) | <0.001 |
| Uric acid, μmol/L | 366.80(105.32) | 352.42(93.11) | 0.018 |
| eGFR, mL/1.73m^2^/min | 64.28(45.37, 87.32) | 77.67(58.36, 100.57) | <0.001 |
| TyG | 8.96(0.56) | 8.96(0.57) | 0.913 |
| TyG group  Q1(<8.67)  Q2(8.67-8.96)  Q3(8.96-9.20)  Q4(>9.20) | 84(25.3)  73(22.0)  96(28.9)  79(23.8) | 668(25.2)  656(24.7)  671(25.3)  660(24.9) | 0.465 |


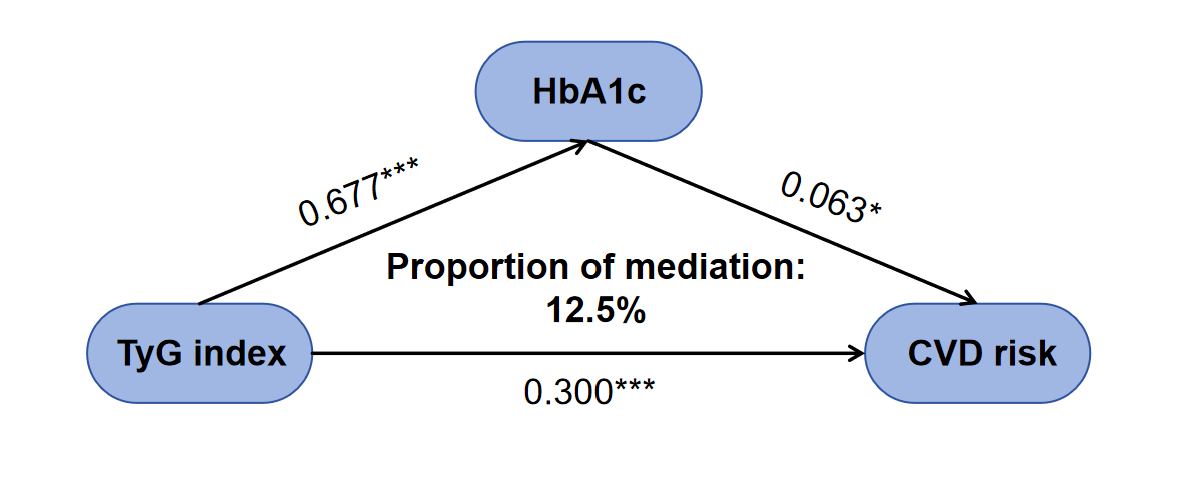
Fig. S1 Mediation analysis of TyG with CVD risk

Adjusted for age, sex, race, educational level, marital status, smoking status, DBP, TC, HDL-C, LDL-C, albumin, eGFR, uric acid, Hypertension, Hypotensive drugs, Chronic kidney disease
